# A nosocomial measles outbreak in Italy, February-April 2017

**DOI:** 10.2807/1560-7917.ES.2017.22.33.30597

**Published:** 2017-08-17

**Authors:** Andrea Porretta, Filippo Quattrone, Francesco Aquino, Giulio Pieve, Beatrice Bruni, Giulia Gemignani, Maria Linda Vatteroni, Mauro Pistello, Gaetano Pierpaolo Privitera, Pier Luigi Lopalco

**Affiliations:** 1 Hygiene and Epidemiology section, Department of Translational Research, New Technologies in Medicine and Surgery, University of Pisa, Pisa, Italy; 2 Medical Direction, Azienda Ospedaliero-Universitaria Pisana, Pisa, Italy; 3 Virology Unit, Pisa University Hospital, Pisa, Italy; 4 Retrovirus Center and Virology Section, Department of Translational Research, New Technologies in Medicine and Surgery, University of Pisa, Pisa, Italy

**Keywords:** measles, healthcare-associated infections, outbreaks, infection control, measles-mumps-rubella vaccine, MMR, vaccine-preventable diseases

## Abstract

We describe a nosocomial outbreak of measles that occurred in an Italian hospital during the first months of 2017, involving 35 persons and including healthcare workers, support personnel working in the hospital, visitors and community contacts. Late diagnosis of the first case, support personnel not being promptly recognised as hospital workers and diffusion of the infection in the emergency department had a major role in sustaining this outbreak.

Measles vaccination coverage is suboptimal in Italy (ranging between 85.4% and 90.6% for one dose during the period 2007–2016) [1], which has led to large pockets of susceptible adults. We describe an outbreak in an Italian hospital between February and April 2017 among healthcare workers (HCW), hospital support personnel (hospital workers in activities not involving direct contact with patients), hospital visitors and community contacts. This outbreak was part of a wider epidemic in Italy that started in January 2017 and has, as at 30 July 2017, led to 4,001 cases nationwide (275 in HCW) [2].

## Outbreak description

A HCW was referred to the emergency department (ED) of an Italian hospital for a rash developed after taking antibiotics for a mild cough and coryza. The rash was considered an allergic reaction to the antibiotic and the HCW was admitted to the hospital inpatient clinic. Three days later, the HCW’s child was admitted to hospital for a rash diagnosed as due to a non-communicable systemic disease.

Subsequent serology showed that both the HCW and the child had measles. The origin of the infection for the index case is still unknown. It should be noted, however, that at least 10 community cases of measles occurred in the same period in the area of residence of the HCW.

Using the standard case definition of the European Commission [3], a total of 34 measles cases during the following weeks were identified by tracing the contacts of the index case. Among them, 15 were HCWs, five were support personnel, four were hospital visitors and 11 were community contacts of the above cases.

The secondary cases to the index (n = 8) occurred in two of the HCW’s relatives, in four other HCWs, and in two support workers who were not immediately recognised as belonging to the hospital outbreak. One of the secondary cases, a HCW, was in service until the onset of symptoms, accessing all rooms on two inpatient wards.

Tertiary cases (n = 6) involved the family of this HCW, three HCWs and a member of support personnel.

Two weeks after the admission of the index case, two of the HCWs belonging to tertiary cases presented during the night to the ED where they stayed for nearly 10 hours. An additional 15 cases could be traced following this single exposure window, namely seven HCWs, two support workers, three relatives of theirs and three visitors to the ED during the time the two HCWs were present. One more community case was related to contact outside hospital with one of the tertiary HCW cases.

Additional cases were related to a visitor at one of the involved inpatient wards, who probably got in contact with one of the tertiary cases who were HCW during the incubation period and generated three cases in the community, among them a family paediatrician. 

**Figure 1:**
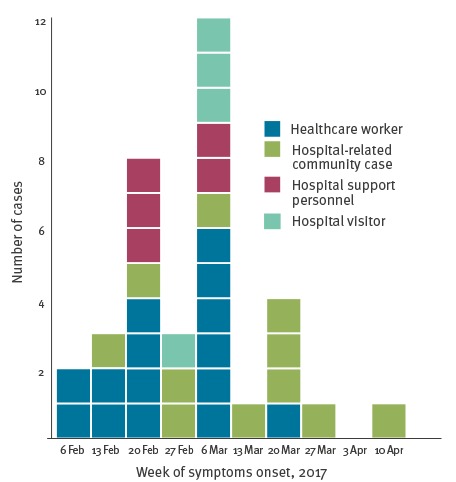
Confirmed measles cases by week of symptom onset and role in hospital setting, nosocomial outbreak, Italy, 5 February–13 April 2017 (n = 35)

**Figure 2:**
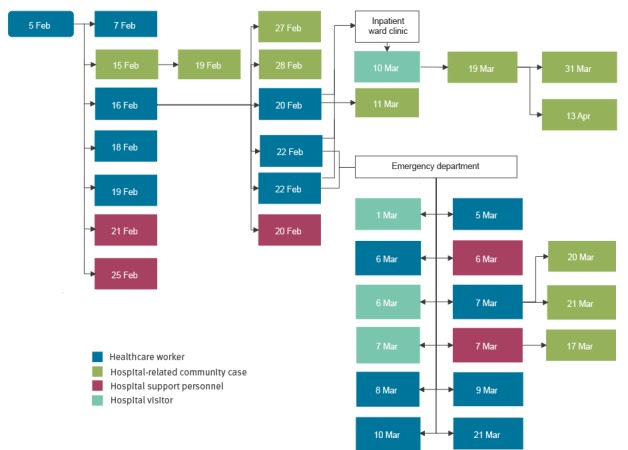
Measles nosocomial outbreak tree with date of symptom onset and role in hospital setting, nosocomial outbreak, Italy, 5 February–13 April 2017 (n = 35)

Figure 1 and Figure 2 show the epidemic curve and the outbreak tree.

## Characteristics of cases

**Figure 3:**
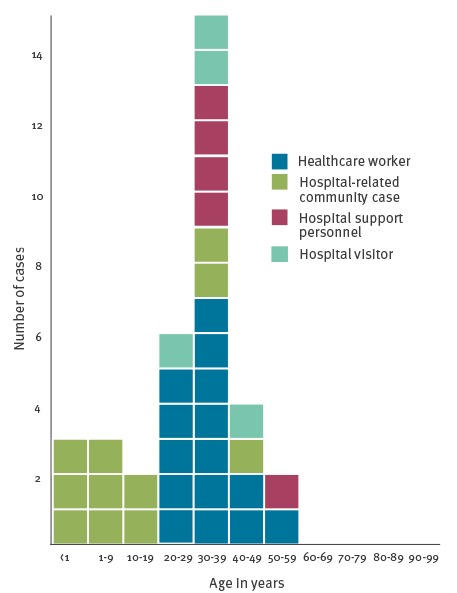
Confirmed measles cases by age group and role in hospital setting, nosocomial outbreak, Italy, 5 February–13 April 2017 (n = 35)

Six cases occurred in children up to nine years of age, while two occurred in adolescents between 10 and 17 years and the majority of cases (n = 27) in adults 18 years and older. Figure 3 describes the age distribution among different categories of cases.

Vaccination status was known for 24 cases. Sixteen were unvaccinated (two children, 14 adults), two had received one dose, one a full course of two doses, and five cases occurred in contacts who received one dose of post-exposure vaccination. Of the five contacts who received post exposure vaccine, one was an infant younger than 1 year for whom information about the date of vaccination is not available; for the other four, the delay between presumed contact date and immunisation was 3 days in two cases, 6 and 12 days for the remaining two cases. Among the HCWs, two were vaccinated, 11 were unvaccinated and for two of them the data was unavailable. Among the five cases belonging to support personnel two were unvaccinated and the status was unknown for the remaining three.

Genotyping was available for three cases: genotype B3, subtype 3.1 was found in all of them.

## Control measures

For each case recorded in the hospital, exposed contacts among personnel and patients were identified and offered a post-exposure vaccination. The Hospital Occupational Health Unit reviewed the immunisation status of all the personnel of the involved units, with an active offer of vaccination for those who were negative. At present, data on the results of this intervention cannot be provided and will be described in a further publication from our occupational health unit. An internal hospital procedure was issued, mandating that all cases with measles-like symptoms were to be assessed in the infectious diseases unit directly rather than passing through the ED.

Regular communication about the epidemiological investigation between the local health authority and the hospital’s epidemiology unit was established in order to share information about all cases occurring in the community. This allowed identification of additional cases among hospital visitors.

## Conclusion and recommendations

Measles elimination in Europe, despite the immunisation efforts, is still jeopardised by recurrent outbreaks in susceptible populations [4-7]. Nosocomial transmission of measles is an important and emerging way of spreading the infection [8-11]. Anyone staying in the hospital environment, regardless of role, can be affected because measles is highly contagious and persists in the environment for up to 2 hours, thus requiring appropriate and timely infection control measures [12,13].

Considering that transmission may occur 3 days before the onset of rash, early diagnosis when only non-specific preliminary symptoms (cough, coryza and conjunctivitis) are present is crucial for containing the outbreak [14].

A single exposure window, that occurred two weeks after the admission of the index case, when two cases were present in the ED, resulted in further 15 cases. Appropriate procedures are needed for patients with suspect transmissible infection in the ED, an issue not limited to measles but shared with several other highly infective conditions [13].

A two-dose vaccination is the most effective measure to prevent measles [15]. This is of crucial importance for HCWs, with a view to their higher risk of exposure and of transmission to vulnerable patients.

However, it should be noted that in at least five cases in this outbreak, measles occurred in personnel working in the hospital environment in support functions, highlighting the need to take into consideration the role of such personnel in the spread of the infection. Given that most of the support personnel belong to outsourced services, coordination is needed between occupational health unit and occupational health responsible of outsourced services.
